# Autism spectrum disorder: communicative profile before and after remote family guidance

**DOI:** 10.1590/2317-1782/e20240238en

**Published:** 2025-10-17

**Authors:** Leonara Emanuelle Honório Silva, Denise Brandão de Oliveira e Britto

**Affiliations:** 1 Departamento de Fonoaudiologia, Faculdade de Medicina, Universidade Federal de Minas Gerais – UFMG - Belo Horizonte (MG), Brasil.

**Keywords:** Autistic Spectrum Disorder, Child Language, Language Development, Communication, Mentoring, Parenting, Speech, Language and Hearing Sciences

## Abstract

**Purpose:**

To compare the communicative profile of children diagnosed with or at risk for autism spectrum disorder before and after speech-language-hearing guidance (indirect treatment).

**Methods:**

The study included caregivers and/or parents of children aged 2 to 9 years with a diagnosis of or at risk for autism spectrum disorder, with or without speech-language-hearing therapy. Before the intervention, caregivers answered the sample characterization form and clinical history. They also sent a 10-minute audio and video recording of interaction between the child and a familiar adult for pragmatic analysis based on the ABFW Child Language Test. The intervention included online guidance meetings with slides and guidance booklets. After the intervention, a new video of child-adult interaction was collected for pragmatic analysis.

**Results:**

There was a statistically significant increase in the number of communicative acts per minute, the number of communicative functions used, and the communicative space occupied by the children from before to after the intervention. Most children changed their preferred means of communication and increased the number of responses, although these changes were not statistically significant. After the guidance meetings, most participants reached the age-appropriate number of communicative acts.

**Conclusion:**

Indirect treatment is a good tool to benefit the pragmatic abilities of children with autism spectrum disorder.

## INTRODUCTION

Autism spectrum disorder (ASD) is a neurodevelopmental disorder characterized by restricted and repetitive patterns of behavior, interests, or activities, and persistent deficits in communication and social interaction^(1)^. The initial ASD symptoms are usually recognized in the first years of life and become more pronounced in early childhood and/or the first years of school. These symptoms are typically manifested by children with developmental language delay, accompanied by a lack of social interest or unusual social interactions. These changes have functional consequences for individuals on the spectrum, particularly in learning through social interaction or in contexts with peers^(1)^.

Social impairment (i.e., changes in the pragmatic and interpersonal use of language) is uniformly found in individuals with ASD, posing one of the greatest challenges for them^(2)^. These deficits in communication and social interaction result in failures in communicative turn-taking and communicative initiative, limitations in interest in sharing information, alterations in verbal and nonverbal communication skills (e.g., impaired eye contact, gestures, and facial expressions), and alterations in the ability to develop and maintain interpersonal relationships in multiple contexts. Deficits in communicative behaviors used for social interaction also result in language changes that may include complete absence of speech, language delay, reduced comprehension, and echoing speech^(1)^.

Consequently, even if children with ASD have adequate communicative competence in vocabulary, syntactic, phonological, or morphological skills, they will not necessarily have efficient communication, since the speech must be consistent with the speaker's intention and coherent with the communicative context^(3)^.

These conditions have a significant impact on the lives of individuals diagnosed with ASD, since the ability to understand language in a social context and respond appropriately to the interlocutor is essential for communication^(1-3)^. Therefore, individual intervention plans for children with ASD must include a professional skilled in stimulating communication, focusing on their pragmatics.

Speech-language-hearing (SLH) pathologists are the professionals responsible for evaluating and intervening in human communication disorders. Therefore, they are essential to promote adequate language development and social interactions in individuals with ASD, as this is one of the greatest challenges faced by such patients. SLH pathologists act as facilitators in the process of identifying symptoms and providing early intervention for changes in communication and social interaction^(4)^.

SLH interventions can be performed directly and indirectly^(4)^. Indirect SLH intervention primarily aims to develop strategies in partnership with family members and caregivers to optimize the patient’s therapy. Indirect ASD intervention can be an extremely valuable tool for prognosis, since family members are the people most aware of the child's development and are the babies' first interlocutors, introducing them to the world and exposing language-facilitating strategies^(5,6)^. Studies describe the benefits of family involvement in facilitating language and communication in children with ASD^(7-9)^. Thus, family members and caregivers must be able to detect atypical manifestations in development and intervene effectively in hindrances encountered in different communicative contexts. However, some caregivers feel challenged when it comes to understanding the set of factors involved in communicative skills^(10)^.

The researchers hypothesized that indirect SLH intervention, delivered through guidance to caregivers of children with ASD, would help to improve caregiver-child interactions. Consequently, it was inferred that these children would improve their social communication skills with appropriate guidance from caregivers, enhancing their functional use of language (the pragmatic subsystem).

Hence, this study aimed to compare the communicative profile of children diagnosed with or at risk of ASD before and after SLH guidance (indirect treatment).

## METHODS

This longitudinal, comparative study analyzed data collected before and after SLH guidance provided to parents and/or caregivers of children with self-reported risk or diagnosis of ASD. It was conducted remotely via the Zoom platform, after approval by the Research Ethics Committee (CEP) of the Federal University of Minas Gerais (UFMG), CAAE: 46948719.7.0000.5149.

Data collection used the following instruments: (1) Structured sample characterization form with information such as name, age, sex, place of residence, suspected diagnosis, and clinical history, and (2) ABFW Child Language Test – pragmatics test^(10)^.

The study was conducted in three stages, as described below. It was publicized in various departments and outpatient clinics through social media and the institution's website. All interested parties received detailed information about the research remotely and signed an informed consent form online via Google Forms. Then, data were collected using a structured sample characterization form and the participants' clinical history (age, sex, education, current and previous SLH therapy, and suspected diagnosis of the child; age, education, and occupation of the parents; sex, education, and family relationship of the caregiver participating in the meetings). The pragmatics test of the ABFW Child Language Test^(11)^ was administered to determine the communicative profile of children with ASD included in the research project. For pragmatic analysis, the family was asked to provide a 10-minute audio and video recording of an interaction between the child and a familiar adult, in a playful situation of habitual communication (adult-child interaction). They were instructed to record the audio and video in a moment of routine interaction with the child for at least 10 consecutive minutes (without cuts or edits). The family was also asked to be the center of the recording, the interaction should occur naturally (as the family normally communicated in daily life), and no children other than the one undergoing intervention should be included in the recording.

Five monthly meetings were held in the second phase to provide guidance to parents and/or caregivers, forming six caregiver groups, each with approximately 20 participants. The objectives, meeting themes, materials used and presented, and the data collection process were the same for all groups.

The guidelines were provided orally by an SLH pathologist and researcher with expertise in ASD, who supervised the study, and by an undergraduate SLH student through online 2-hour meetings. They shared slides and guidance booklets (support material) made available digitally, as proposed by Fernandes^(12)^.

The first meeting’s theme was “The context”, highlighting approaches to the family context, the role of caregivers, the value of child communication, the need to stimulate language in the child's natural context, and the importance of paying attention to sensory issues of individuals with ASD.

The second meeting, “How and Why Children Can Communicate,” provided notions about communication, speech, and language, the recognition of different communicative means and functions, and clarifications on how to increase the child's eye contact and communicative initiatives and possibilities.

“Active engagement” was the theme of the third meeting, which discussed the importance of valuing children's spontaneous actions in each context and the importance of possessing skills to improve their participation in different situations. Explanations were also provided regarding skills and aspects considered prerequisites for good communicative performance (joint attention, self-regulation, emotional and physical availability, attentiveness, and turn-taking).

The fourth meeting approached aspects that influence communication, highlighting both positive and negative aspects that impact listening and speaking skills. Furthermore, strategies were presented to promote language development, work on nonverbal aspects of communication, expand communicative functions and their effective use, adapt communicative means for more efficient use, stimulate cognitive functions, and expand receptive and expressive vocabulary. The importance of motivation and engagement in play was also highlighted at this meeting.

“Expanding Communication Opportunities” was the theme of the fifth and final guidance meeting. It addressed the importance of promoting strategies to enhance communication and encouraged caregivers to create routines according to the child's preferences, relating them to quality of life and social engagement. It also emphasized strategies aimed at expanding discursive possibilities, developing conversational skills, improving socio-communicative and problem-solving skills, and redirecting expressions toward more socially appropriate situations (such as restricted interest repertoire, echolalia, stereotypies, self-aggression, hetero-aggression, and strict routine). In addition to following Fernandes's proposal^(12)^, all five meetings provided space for reflection, questions, and specific demands of participating caregivers so that the offerings also met the specific needs of the group and participants.

In the third stage of the research, participants were asked to submit a new video of adult-child interaction for pragmatic analysis using the ABFW test^(11)^, comparing data before and after the guidance sessions. An undergraduate SLH student properly trained by the study's supervising researcher performed the pragmatic analysis before and after the guidance sessions. It was subsequently reviewed by an SLH pathologist with experience in language development. The professional conducting the analyses had no prior relationship with the families or children who received the intervention.

**Figure 1:**
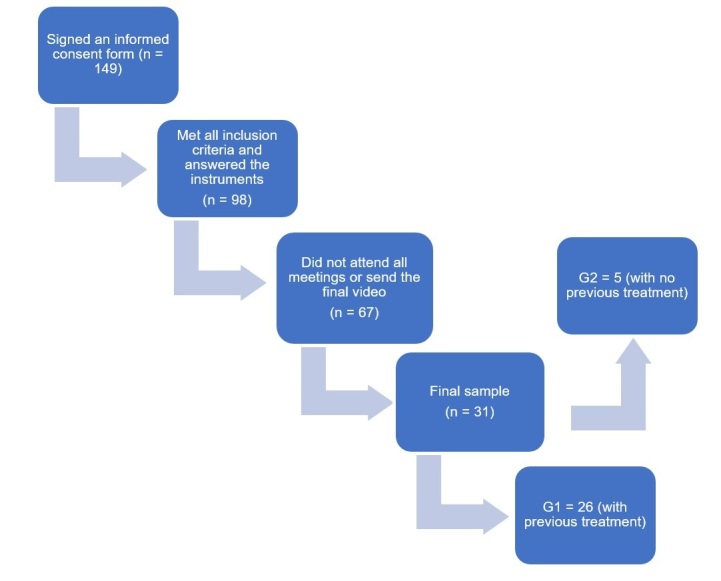
Sample composition

This was a non-probabilistic sampling method, which invited caregivers and/or parents of children with a reported diagnosis or risk of ASD, with or without SLH support, who were interested in participating in the study. The inclusion criteria were caregivers and/or parents of children aged 2 to 9 years with a diagnosis or risk of ASD, with or without SLH support, who agreed to respond to the questionnaires and film the necessary material for pragmatic analysis according to the ABFW protocol, and signed an informed consent form. The exclusion criteria were caregivers and/or parents of children who withdrew from the study, who missed two or more of the five guidance meetings, and who did not submit the final interaction video after the five guidance meetings (Figure 1).

Descriptive data analysis was performed to achieve the study objective, using the frequency distribution of categorical variables and analysis of measures of central tendency and dispersion of continuous variables. The results were divided into two groups: G1, whose participants had undergone or were undergoing SLH therapy, and G2, whose participants had never undergone SLH therapy.

The Wilcoxon and McNemar tests were used for association analyses to verify the pre-meeting and post-meeting results. Significance was set at the p-value of 0.05.

SPSS software, version 25.0, was used for data entry, processing, and analysis.

## RESULTS

The total sample consisted of caregivers of 31 children, of whom 26 (G1) had undergone or were undergoing SLH therapy at the time of the study, and five (G2) were not undergoing and had never undergone direct intervention/SLH therapy. The mean age of the children who had undergone direct intervention at the beginning of the meetings (G1) was 4.56 years, with a standard deviation of 2.10 and a median of 3.75 years. At the end of the meetings, their mean age was 4.95 years, with a standard deviation of 2.06 and a median of 4.11. The mean age of G2 at the beginning of the meetings was 3.44 years, with a standard deviation of 1.58 and a median of 3.00 years; at the end, their mean age was 3.72 years, with a standard deviation of 1.70 and a median of 3.30.

The analysis of G1’s demographic and characterization data revealed that most children were males (65.4%), attended preschool (73.1%), and had a diagnosis of ASD (84.6%). Most parents had a bachelor’s degree (76.9% of mothers and 50.0% of fathers), and half of the mothers and fathers were employed (50.0% each). Most caregivers who participated in the guidance meetings were females (92.3%), mothers of the children (90.3%), had a bachelor’s degree (76.9%), and participated in all meetings (67.7%). G2’s demographic and characterization data revealed that most children were males (80.0%), attended preschool (60.0%), and had a suspected diagnosis of ASD (60.0%). Most parents had a bachelor’s degree (60.0% for both) and were employed (60.0% each). All caregivers were females, the children's mothers (100.0%), and the majority had a bachelor’s degree (60.0%) (Table 1).

**Table 1:** Descriptive analysis of demographic data and sample characterization

**Variables**	**G1 - 26**		**G2 - 5**	
	**N**	**%**	**N**	**%**
**Child’s sex**				
Females	9	34.6	1	20.0
Males	17	65.4	4	80.0
Total	26	100.0	5	100.0
**Child’s education**				
Not attending school	3	11.5	1	20.0
Preschool	19	73.1	3	60.0
Elementary school	4	15.4	1	20.0
Total	26	100.0	5	100.0
**ASD diagnosis**				
No	4	15.4	3	60.0
Yes	22	84.6	2	40.0
Total	26	100.0	5	100.0
**Mother’s education**				
Middle school Incomplete	1	3.8	-	-
High school graduate	5	19.2	2	40.0
Bachelor’s degree	20	76.9	3	60.0
Total	26	100.0	5	100.0
**Mother’s occupation**				
Domestic workers	8	30.8	-	-
Self-employed	5	19.2	2	40.0
Employed	13	50.0	3	60.0
Total	26	100.0	5	100.0
**Father’s education**				
Middle school Incomplete	1	3.8	1	20.0
High school incomplete	1	3.8	1	20.0
High school graduate	8	30.8	-	-
Higher education incomplete	3	11.5	-	-
Bachelor’s degree	13	50.0	3	60.0
Total	26	100.0	5	100.0
**Father’s education**				
Self-employed	10	38.5	2	40.0
Employed	13	50.0	3	60.0
Unemployed	3	11.5		
Total	26	100.0	5	100.0
**Caregiver’s sex**				
Males	2	7.7	0	0.0
Females	24	92.3	5	100.0
Total	26	100.0	5	100.0
**Caregiver’s education**				
Middle school incomplete	1	3.8	-	-
High school graduate	5	19.2	2	40.0
Bachelor’s degree	20	76.9	3	60.0
Total	26	100.0	5	100.0
**Caregiver’s family relationship**				
Mother	28	90.3	5	100.0
Father	2	6.5	0	0.0
Grandmother	1	3.2	0	0.0
Total	31	100.0	5	100.0
**The caregiver attended all meetings**				
No	10	32.3	3	60.0
Yes	21	67.7	2	40.0
Total	31	100.0	5	100.0

**Caption:** N = number of individuals

Descriptive measures of the communicative profile of children who were and were not in direct therapy were analyzed using the ABFW Pragmatics test before and after the sessions. Children in G1 (those who had undergone or were undergoing SLH therapy) had a mean of 5.09 communicative acts per minute before the sessions and 7.15 after them, and a mean of 6.92 communicative functions before the sessions and 8.73 after them. The study also counted the number of children’s responses (not identifying communicative functions), revealing a mean of 23.12 before the sessions and 25.65 after them. The mean communicative space occupied by the children was 24.77% and 31.58% before and after the meetings, respectively. G2’s descriptive analysis found that the mean number of communicative acts per minute was 5.04 before the meetings and 6.86 after them. The number of communicative functions used did not change, with a mean of 8.60 before and after the meetings. The children’s mean number of responses (not identifying communicative functions) was 28.60 before the meetings and 18.80 after them. The mean communicative space occupied by these children was 25.60% and 40.60% before and after the meetings, respectively.

The following data were recognized to complete the descriptive analysis of the children's communicative profile assessment before and after the intervention (ABFW Pragmatics test): most children did not have the number of communicative acts expected for their age^(11)^ before the meetings (76.9% of children who had undergone or were undergoing therapy and 80.0% of those who had never undergone direct intervention). After the guidance meetings, most participants achieved the number of communicative acts appropriate for their age (53.8% of children in G1 and 80.0% of children in G2). Most children in direct intervention preferentially used gestural means to communicate before the meetings (38.5%). In contrast, most of them preferentially used verbal means (48.3%) for communication after the meetings (Table 2).

**Table 2:** Descriptive analysis of the communicative profile assessment before and after meetings

**Variables**	**G1 - 26**			**G2 - 5**					
	**Before After Before After**								
	**N**	**%**	**N**		**%**	**N**	**%**	**N**	**%**
**Number of age-appropriate communicative acts**									
No	20	76.9	12		46.2	4	80.0	1	20.0
Yes	6	23.1	14		53.8	1	20.0	4	80.0
**The most used means of communication**									
Verbal	8	30.8	11		42.3	3	60.0	4	80.0
Vocal	8	30.8	8		30.8	0	0	0	0
Gestural	10	38.4	7		26.9	2	40.0	1	20.0

**Caption:** N = number of individuals

The association analysis between communicative acts per minute, number of communicative functions used, number of responses, and percentage of communicative space used by G1 children found a statistically significant result before and after guidance meetings between communicative acts per minute (p = 0.002), with more communicative acts per minute after the meetings; number of communicative functions used (p = 0.016), with more functions after the meetings; communicative space (p = 0.001), with a greater communicative space used by the children after the meetings. In G2, the association analysis between communicative acts per minute, number of communicative functions used, number of responses, and percentage of communicative space used revealed a statistically significant result before and after guidance meetings between communicative acts per minute (p = 0.042), with more communicative acts per minute after the meetings. The other associations were not statistically significant (Table 3).

**Table 3:** Association analysis of communicative functions before and after meetings

**G1 – 26 G2 - 5**						
**Variables**	**Communicative functions**					
	**Before**	**After**	**p-value**	**Before**	**After**	**p-value**
**Communicative acts/minute**						
Mean±SD	5.09±2.93	7.15±2.58	0.002*	5.04±1.61	6.86±1.60	0.042*
Median	4.35	6.80		5.10	7.60	
**Number of functions used**						
Mean±SD	6.92±2.61	8.73±2.07	0.016*	8.60±2.97	8.60±2.88	0.785
Median	7.00	9.00		9.00	10.00	
**Number of responses**						
Mean±SD	23.12±22.17	25.65±16.57	0.271	28.60±19.14	18.80±19.64	0.500
Median	15.00	21.50		31.00	11.00	
**Communicative space**						
Mean±SD	24.77±9.59	31.58±7.45	0.001*	25.60±7.89	40.60±9.81	0.104
Median	23.50	32.00		26.00	37.00	

Wilcoxon test

* p-value ≤ 0.05

**Caption:** SD = standard deviation

## DISCUSSION

The comparison of communication profiles of children with ASD before and after SLH therapy found a statistically significant increase in the number of communicative acts per minute in both children receiving direct intervention and those who had never received SLH therapy. Those receiving SLH therapy at the time of the study had a statistically significant increase in the number of communicative functions used and the communicative space occupied when receiving the indirect intervention offered in this study.

The increase in the number of communicative acts per minute is consistent with an improvement in the participants' pragmatic profile and, therefore, an improvement in social communication. This finding corroborates another study focusing on parental guidance to train parents of children with ASD to use everyday situations for language acquisition^(13)^. It reports that the increase in the number of communicative acts per minute after guidance enabled most children to achieve an age-appropriate number of communicative acts, revealing an improvement in the pragmatic impairments found before the intervention.

The increase in the number of communicative functions used by children undergoing SLH therapy after receiving guidance from caregivers also implies an improvement in the participants' pragmatic skills. This finding is supported by another study^(14)^, which reports that pragmatic impairment in ASD involves disadvantages in the communicative functions acquired and used by individuals, especially interpersonal functions, causing interactive difficulties for the population. Another study^(3)^ corroborates the present one, observing that children with ASD increased their flexibility in the use of communicative functions after an SLH therapy program. Thus, these studies highlight the importance of indirect treatment for variability in using communicative language functions^(3)^ and the consequent improvement in the children's pragmatic profile.

Another finding in the sample of children in this study was the increase in the communicative space occupied by them after the guidance sessions, a statistically significant increase for children who were in direct intervention at the time of the study. This finding is closely related to the increase in the number of communicative acts per minute and the reduction in conversational monopolization, the number of commands given to the child, and the bombardment of information after the treatment. It is noteworthy that children who had never been in direct intervention also increased their communicative space, although this finding was not statistically significant. Children with typical language development maintain dialogue by responding and initiating conversations efficiently, and they rarely fail to occupy the communicative space, according to a study^(15)^, which favors the maintenance of interaction. It can be inferred that the guidance sessions helped parents to understand the importance of paying attention to social reciprocity and seeking symmetrical communicative space during interactions with their children, awaiting the child's spontaneous expressions. A previous study^(16)^ observed an improvement in linguistic performance between mothers and children after the SLH guidance to wait for the child's expressions and to value moments of pause and silence. Thus, the emphasis given to moments of silence allowed the interaction to occur more fluidly, since objective questions were reduced, and the child's interest was valued.

The descriptive analysis highlighted issues related to sex, since most children in the study were males. According to the Diagnostic and Statistical Manual of Mental Disorders (DSM-5), ASD is four times more frequently diagnosed in male children, corroborating the prevalence of boys in this study^(1)^.

This study demonstrated that most participants had access to direct SLH therapy at the time of the study. Important advances have been made in recent years for individuals with ASD, such as the creation of Psychosocial Care Centers dedicated to treating individuals with autism^(17)^. Therefore, it can be inferred that the number of children receiving treatment, including SLH therapy, has also increased, and that there has been an improvement in access to therapy for children with ASD, agreeing with the data found in this study.

Most caregivers who participated in the guidance meetings were females, which may be explained by the prevalence of women seeking healthcare compared to men^(18)^. Research indicates that women perceive their health status more negatively and report more morbidities than men, which explains their greater need for healthcare^(19-21)^. Furthermore, this study showed that most meeting participants were mothers of the children, as in other studies involving the participation of parents and caregivers of children with ASD^(3,12)^. Therefore, the present study agrees with the previous ones, highlighting greater maternal participation in the children's therapeutic process when compared to other caregivers.

It can be inferred that the fact that most caregivers had a bachelor’s degree and participated in all meetings positively influenced the increase in the number of communicative acts per minute, the communicative space, and the number of communicative functions used after the intervention. According to the research literature, some studies^(22,23)^ suggest that parental education interferes with the early identification of ASD signs. In other words, the higher the parents' education level, the earlier the signs of ASD are observed; likewise, lower parental education levels contribute to delayed diagnoses. Hence, it can also be inferred that caregivers seek treatment earlier when symptoms are identified early. Furthermore, the vast majority of children in the study had a confirmed diagnosis of ASD, which relates to the studies previously cited^(22,23)^, demonstrating an earlier identification of ASD symptoms by parents with higher education levels.

The descriptive analysis of clinical data found that the number of responses given by children who had never received SLH therapy decreased, while the responses by children who received direct intervention increased, though not significantly. Communicative acts produced by children whose sole function was to respond to questions and commands provided by the interlocutor were considered “responses,” since these acts did not have any other communicative function described in the instrument used for analysis^(11)^. The reduction in the number of responses in the group of children without direct intervention and the non-statistically significant association among children receiving direct SLH therapy when comparing the number of responses before and after the intervention can be explained by the fact that parents were instructed to reduce the number of questions and commands to their children. This reduction aimed to expand their children's communicative initiatives and improve the symmetry of the communicative space. Thus, parents were instructed to replace questions and commands with comments and naming. Moreover, besides these instructions, parents were asked to wait for the child's linguistic processing when asking questions, so they could develop their own answers, instead of answering for the child^(16)^. Thus, it can be inferred that the parents sought to follow both instructions, possibly interfering with the results in the “number of answers given by the child”.

Regarding the total sample’s communicative means, most children in direct SLH therapy changed their preferred means of communication (predominant gestural means before the intervention to predominant verbal means after it), although this change was not statistically significant. However, the increase in the use of verbal means of communication is extremely valuable, since it indicates that the children began to use speech more intensively to communicate after the guidance. Another study likewise found an increased use of verbal means of communication after parental guidance; participating parents reported noticing that their children increased verbalization to express both interpersonal and non-interpersonal functions^(3)^.

Hence, this study and the other ones cited demonstrate the importance of including parents and caregivers in the therapeutic process to improve children's linguistic performance in a natural and spontaneous context and reduce their communication difficulties^(24)^. Another study^(25)^ corroborates this statement, noting that parent-mediated social communication intervention is effective in reducing ASD symptoms and promoting lasting effects on social interaction between parents and children. Yet another study^(26)^ observed improvements in the social communication skills of children with ASD and the communicative competence of the parents after a parent training intervention. A third study^(27)^ found an improvement in the social communication skills of children with ASD after parental guidance, once again corroborating the benefits of indirect intervention in the treatment of these children.

The current study demonstrated that indirect intervention combined with direct SLH therapy provides greater benefits to the communication of children with ASD than indirect intervention alone. This finding corroborates another study^(28)^ that demonstrated that SLH pathologists who intervene directly and indirectly with individuals with ASD obtain broader improvements in their linguistic development, since information provided to parents provides better monitoring of children with ASD.

The main limitation of the study concerns the number of participants at the beginning and end of the guidance meetings. At the beginning of the study, 149 parents expressed interest in receiving guidance by signing an informed consent form provided remotely. Ninety-eight caregivers completed the sample characterization and clinical history questionnaire and submitted audio and video recordings. Of these, seven did not participate in any of the guidance meetings. Also, eight of the caregivers who participated in four or more guidance meetings did not submit the audio and video recordings for final analysis, which consequently reduced the data used in the sample. Thus, 68.3% of the participants were not included in the final study sample, 7.1% did not attend any of the guidance meetings, and 8.1% attended the meetings but did not submit the final recording. In other words, 53% of the caregivers withdrew from the study without prior justification. The time allotted for monthly meetings (2 hours each) may have negatively influenced parental participation until the end of the study.

Moreover, the convenience sample and heterogeneous groups prevent generalization of the findings to other contexts and make it impossible to compare children who were and were not receiving SLH therapy. Therefore, we suggest expanding the study to reach more caregivers and provide a comparison group to address these limitations.

One advance achieved by the study is the active involvement of families in the development of children with ASD. Parent training through guidance from professionals is an important means of improving the language skills of children with ASD, since parents spend the most time with these children and are more likely to stimulate them in a natural and spontaneous context.

Furthermore, this study provided parents with a pragmatic perspective focusing on the use of questions and answers during communication and other communicative functions to initiate and maintain functional interactions. SLH therapy for individuals with ASD should focus on the pragmatic subsystem of language, since it is by using language that individuals can maintain conversation and exchange information.

## CONCLUSION

The conclusion of the study is that comparing the communicative profile of children before and after the guidance revealed significant changes, including a statistically significant increase in the number of communicative acts per minute. It also found a statistically significant increase in the number of communicative functions used and the communicative space occupied by children undergoing direct therapy after the indirect intervention described in this study, demonstrating improvements in the participants' social communication. Therefore, SLH guidance (indirect treatment) is a valuable intervention tool that helps improve the pragmatic skills of children with ASD.
