# Supplementary material for: Autism spectrum disorder: communicative profile before and after remote family guidance
Source: Codas. 2025 Oct 17;37(5):e20240238. doi: 10.1590/2317-1782/e20240238en (PMC12533530; doi:10.1590/2317-1782/e20240238en)
Supplement: Translation

# Translation: Transtorno do espectro do autismo: perfil comunicativo antes e após orientação familiar remota

**DOI:** 10.1590/2317-1782/e20240238pt

**Authors:** Leonara Emanuelle Honório Silva, Denise Brandão de Oliveira e Britto

**Affiliations:** 1 Departamento de Fonoaudiologia, Faculdade de Medicina, Universidade Federal de Minas Gerais – UFMG - Belo Horizonte (MG), Brasil.

**Keywords:** Transtorno do Espectro do Autismo, Linguagem Infantil, Desenvolvimento da Linguagem, Comunicação, Tutoria, Parentalidade, Fonoaudiologia

## Abstract

**Objetivo:**

Comparar o perfil comunicativo de crianças com diagnóstico ou risco de Transtorno do Espectro do Autismo antes e após a realização de orientações fonoaudiológicas (tratamento indireto).

**Método:**

Participaram deste estudo cuidadores e/ou pais de crianças de dois a nove anos com diagnóstico, ou risco autorrelatado para Transtorno do Espectro do Autismo, com ou sem acompanhamento fonoaudiológico. Anteriormente a intervenção, os cuidadores preencheram os dados do roteiro de caracterização da amostra e da história clínica. Ademais, os cuidadores enviaram um registro em áudio e vídeo, de dez minutos de interação entre a criança e um adulto familiar para análise pragmática à luz do Teste de Linguagem Infantil ABFW. A intervenção incluiu encontros de orientações on-line com compartilhamento de slides e cartilhas de orientação. Após a intervenção foi realizada a coleta de novo vídeo de interação entre adulto e criança para realização da análise pragmática.

**Resultados:**

Houve um aumento estatisticamente significativo do número de atos comunicativos por minuto, do número de funções comunicativas utilizadas e do espaço comunicativo ocupado pelas crianças no momento pós-intervenção quando comparado ao momento anterior a intervenção. A maioria das crianças modificou seu meio comunicativo utilizado preferencialmente e aumentou o número de respostas dadas, apesar dessas mudanças não serem estatisticamente significativas. Após os encontros de orientação, a maioria dos participantes alcançou o número de atos comunicativos adequado para a idade.

**Conclusão:**

Houve mudanças estatisticamente significativas ao comparar o perfil comunicativo das crianças antes e após as orientações, indicando benefícios nas habilidades pragmáticas dos participantes.

## INTRODUÇÃO

O Transtorno do Espectro do Autismo (TEA) é um transtorno do neurodesenvolvimento que se caracteriza por presença de padrões restritos e repetitivos de comportamento, interesse ou atividades, além de déficits persistentes de comunicação e interação social^(1)^. Os sintomas iniciais do Transtorno do Espectro do Autismo costumam ser reconhecidos nos primeiros anos de vida e acentuam-se na primeira infância e/ou nos primeiros anos de vida escolar. Esses sintomas normalmente são manifestados por crianças com atraso no desenvolvimento da linguagem acompanhado por ausência de interesse social ou interações sociais incomuns. As alterações citadas, geram consequências funcionais para os indivíduos que estão no espectro, principalmente no que diz respeito à aprendizagem por meio da interação social ou em contextos com seus pares^(1)^.

O comprometimento social, ou seja, as alterações nos usos pragmático e interpessoais da linguagem são encontradas de maneira uniforme nos indivíduos com Transtorno do Espectro do Autismo, além de se apresentarem como um dos maiores desafios encontrados para essa população^(2)^. Esses déficits na comunicação e interação social resultam em falhas nas trocas de turno comunicativo e na iniciativa comunicativa, limitações no interesse em compartilhar informações, alterações nas habilidades de comunicação verbal e não verbal como falhas no contato visual, nos gestos e expressões faciais, bem como alterações na capacidade de desenvolver e manter relações interpessoais em múltiplos contextos. Os déficits nos comportamentos comunicativos utilizados para a interação social resultam ainda em alterações de linguagem que variam de ausência total da fala, atraso de linguagem, compreensão reduzida e fala em eco^(1)^.

Em consequência, mesmo se houver uma adequada competência comunicativa em vocabulário, habilidades sintáticas, fonológicas ou morfológicas nas crianças com TEA, esses indivíduos não necessariamente terão uma comunicação eficiente, uma vez que é preciso que o discurso esteja condizente com a intenção do falante e coeso com o contexto comunicativo^(3)^.

As alterações citadas causam efeito significativo na vida dos indivíduos diagnosticados com TEA uma vez que a capacidade de entender a linguagem em contexto social assim como a capacidade de responder ao interlocutor de maneira adequada é imprescindível para a comunicação^(1-3)^. Por conseguinte, torna-se evidente a necessidade de incluir no plano individual de intervenção das crianças com TEA um profissional com competência na estimulação da comunicação, dando enfoque na pragmática do indivíduo.

O fonoaudiólogo é o profissional responsável por avaliar e intervir nos distúrbios da comunicação humana. Diante disso, o fonoaudiólogo se torna fundamental para promover o adequado desenvolvimento da linguagem e consequentemente das interações sociais do indivíduo com Transtorno do Espectro do Autismo, uma vez que esse é um dos maiores desafios enfrentados no TEA. O fonoaudiólogo atua como um facilitador no processo de busca por identificação de sintomas e intervenção precoce nas alterações de comunicação e interação social^(4)^.

As intervenções fonoaudiológicas podem ser realizadas direta e/ou indiretamente^(4)^. A intervenção fonoaudiológica indireta visa, primordialmente, a construção de estratégias em parceria com os familiares e cuidadores para maximizar o processo terapêutico do indivíduo tratado. Para o Transtorno do Espectro do Autismo, a intervenção indireta pode ser uma ferramenta de extrema valia para o prognóstico do caso, uma vez que os membros da família são as pessoas que mais estão a par do desenvolvimento da criança além de serem os primeiros interlocutores dos bebês, responsáveis por apresentar-lhes o mundo e expor estratégias facilitadores da linguagem^(5,6)^. Existem estudos que descrevem os benefícios do envolvimento familiar no processo de facilitação da linguagem e da comunicação de crianças com TEA^(7-9)^. É necessário, portanto, que os familiares e cuidadores estejam aptos para detectar as manifestações atípicas no desenvolvimento, assim como para intervir de maneira eficaz nos impasses encontrados em diferentes contextos comunicativos. Porém, alguns cuidadores se sentem desafiados no que diz respeito a compreensão do conjunto de fatores que envolvem as competências comunicativas^(10)^.

A hipótese das pesquisadoras era de que a intervenção fonoaudiológica indireta, realizada por meio de orientações aos cuidadores de crianças com Transtorno do Espectro do Autismo, contribuiria para melhorar a interação dos cuidadores com as crianças. Em consequência, inferia-se que, com a condução adequada realizada pelos cuidadores, essas crianças aperfeiçoariam suas habilidades comunicativas sociais aprimorando o uso funcional da linguagem, ou seja, o subsistema pragmático.

Destarte, o objetivo do presente estudo foi comparar o perfil comunicativo de crianças com diagnóstico ou risco de Transtorno do Espectro do Autismo antes e após a realização de orientações fonoaudiológicas (tratamento indireto).

## MÉTODO

Realizou-se um estudo longitudinal, comparativo, com análise de dados coletados antes e após orientações fonoaudiológicas realizadas com pais e/ou cuidadores de crianças com risco autorrelatado, ou diagnóstico de TEA. O estudo foi realizado de maneira remota por meio da plataforma Zoom, após aprovação do Comitê de Ética em Pesquisa (CEP) da Universidade Federal de Minas Gerais (UFMG), CAAE: 46948719.7.0000.5149.

Foram utilizados os seguintes instrumentos para coleta de dados: (1) Roteiro estruturado de caracterização da amostra contendo informações como nome, idade, sexo, local de moradia e hipótese diagnóstica, além de dados da história clínica e (2) Teste de Linguagem Infantil ABFW – prova de pragmática^(10)^.

O estudo foi realizado em três etapas conforme se descreve. A divulgação do estudo foi realizada por meio de redes sociais, nos diferentes departamentos, ambulatórios e no site da instituição. Todos interessados receberam de maneira remota síncrona esclarecimentos detalhados acerca da pesquisa e realizaram a assinatura do Termo de Consentimento Livre e Esclarecido (TCLE) on-line via *Google Forms*. Em seguida, foram coletados dados por meio do roteiro estruturado de caracterização da amostra e dados da história clínica dos participantes (idade, sexo, escolaridade, tratamento fonoaudiológico atual e pregresso e hipótese diagnóstica da criança; idade, escolaridade e profissão dos pais; sexo, escolaridade e posição familiar do cuidador participante dos encontros). Visando determinar o perfil comunicativo das crianças com TEA inclusas no projeto de pesquisa, foi aplicada a prova de pragmática do Teste de Linguagem Infantil ABFW^(11)^. Para análise pragmática, solicitou-se à família, um registro em áudio e vídeo, de dez minutos de uma interação entre a criança e um adulto familiar, em uma situação lúdica de comunicação habitual (interação adulto e criança). Para a coleta do registro em áudio e vídeo instruiu-se a família a gravar um momento de interação corriqueira com a criança, por no mínimo 10 minutos sequenciais (sem cortes ou edições). Solicitou-se ainda que o adulto e a criança estivessem ao centro da filmagem e que a interação ocorresse de forma natural (como normalmente a família comunicava-se no dia a dia). Instruiu-se também que outras crianças não fossem incluídas na filmagem, que não a criança alvo da intervenção.

Durante a segunda etapa foram organizados cinco encontros mensais para orientações dos pais e/ou cuidadores. Foram organizados seis grupos de cuidadores com cerca de 20 participantes cada. O objetivo, temas dos encontros, materiais utilizados e apresentados foram os mesmos para todos os grupos, assim como o processo de coleta de dados.

As orientações foram realizadas oralmente por uma profissional fonoaudióloga, pesquisadora orientadora do trabalho, com expertise em Transtorno do Espectro Autista e por uma aluna de graduação do curso de fonoaudiologia por meio de encontros on-line de duas horas cada, com compartilhamento de *slides* e cartilhas de orientação (material de suporte) disponibilizadas digitalmente seguindo a proposta de Fernandes^(12)^.

O primeiro encontro possuiu como tema “O contexto”, no qual se destacaram abordagens acerca do contexto familiar, papel dos cuidadores, valorização da comunicação da criança, necessidade de estimulação da linguagem no contexto natural da criança e importância de atentar-se às questões sensoriais dos indivíduos com TEA.

No segundo encontro “Como e por que as crianças podem se comunicar”, abordou-se o fornecimento de noções sobre a comunicação, fala e linguagem; o reconhecimento dos diferentes meios e funções comunicativas em adição a esclarecimentos sobre como ampliar contato visual, aumentar as iniciativas comunicativas e ampliar as possibilidades comunicativas da criança.

O “Engajamento ativo” foi tema do terceiro encontro, no qual se falou sobre a importância de valorizar os atos espontâneos da criança em determinado contexto e a relevância de possuir habilidades para melhorar a participação da criança em diferentes situações. Forneceram-se ainda explicações a respeito de habilidades e aspectos considerados pré-requisitos para um bom desempenho comunicativo (atenção compartilhada, autorregulação, disponibilidade emocional e corporal, atenção e troca de turnos).

No quarto encontro, destacaram-se os aspectos que influenciam na comunicação, expondo aspectos positivos e negativos que interferem nas capacidades de ouvir e falar. Para mais, houve apresentação de estratégias visando favorecer o desenvolvimento da linguagem, trabalhar aspectos não-verbais da comunicação, ampliar as funções comunicativas e seu uso efetivo, adequar meios comunicativos para sua utilização mais eficiente, estimular funções cognitivas e ampliar vocabulário receptivo e expressivo. A importância da motivação e do envolvimento com o brincar também ganhou destaque nesse encontro.

“Ampliando as oportunidades comunicativas” foi tema do quinto e último encontro de orientações. Expôs-se a importância da promoção de estratégias que visam ampliar a comunicação, além de fornecer encorajamento aos cuidadores para a criação de rotinas. Apontou-se aos ouvintes que a rotina deve ser elaborada conforme as preferências da criança relacionando-as com a qualidade de vida e envolvimento social. Ademais, foram ressaltadas estratégias objetivando ampliar as possibilidades discursivas, desenvolver habilidades conversacionais, aperfeiçoar habilidades sociocomunicativas e resolução de situações-problema, além de redirecionar manifestações para situações mais socialmente adequadas (tais como repertório de interesses restritos, ecolalias, estereotipias, auto e heteroagressão e rigidez de rotina). Nos cinco encontros, além de seguir a proposta de Fernandes^(12)^, foram mantidos espaços para reflexões, questionamentos e demandas específicas dos cuidadores participantes para que a oferta atendesse também as peculiaridades do grupo e dos participantes.

Na terceira etapa da pesquisa, foi solicitado aos participantes o envio de novo vídeo de interação adulto e criança para realização da análise pragmática por meio da prova do ABFW^(11)^, a fim de comparar os dados antes e após as orientações. A análise pragmática realizada antes e após a realização dos encontros de orientação foi realizada por uma estudante de graduação do curso de Fonoaudiologia, devidamente treinada pela pesquisadora orientadora do estudo, e posteriormente revisada por um profissional fonoaudiólogo com experiência em desenvolvimento da linguagem. O profissional responsável por realizar as análises não possuía vínculo prévio ao estudo com as famílias e/ou crianças que receberam a intervenção.

Trata-se de amostragem não probabilística, na qual cuidadores e/ou pais de crianças com relato de diagnóstico, ou risco para Transtorno do Espectro do Autismo, com ou sem acompanhamento fonoaudiológico, que se interessaram em participar do estudo, foram convidados. Os critérios de inclusão foram: cuidadores e/ou pais de crianças de dois a nove anos com diagnóstico, ou risco para Transtorno do Espectro do Autismo, com ou sem acompanhamento fonoaudiológico, que concordaram em responder aos questionários e em realizar as filmagens necessárias para análise pragmática conforme o protocolo do ABFW, além da assinatura do Termo de Consentimento Livre e Esclarecido (TCLE). Como critérios de exclusão cita-se: cuidadores e/ou pais de crianças que desistiram de participar do estudo, cuidadores e/ou pais com duas ou mais faltas nos cinco encontros de orientação, cuidadores e/ou pais que não enviaram o vídeo de interação final, após os cinco encontros de orientação (Figura 1).

**Figura 1:** Composição da amostra

Visando atender ao objetivo do estudo foi realizada a análise descritiva dos dados, por meio da distribuição de frequência das variáveis categóricas e análise das medidas de tendência central e de dispersão das variáveis contínuas. Os resultados foram divididos em dois grupos, a saber: G1 composto por participantes que estavam ou estiveram em terapia fonoaudiológica anteriormente e G2, grupo de participantes que nunca realizaram terapia fonoaudiológica.

Para as análises de associação, visando verificar os resultados pré e pós-encontros foram utilizados os testes Wilcoxon e McNemar, sendo considerados resultados significativos os que apresentaram valor de p≤0,05.

Para entrada, processamento e análise dos dados foi utilizado o software SPSS, versão 25.0.

## RESULTADOS

A amostra total foi composta por cuidadores de 31 crianças. Dentre estes, 26 crianças (G1) haviam realizado ou estavam em terapia fonoaudiológica no momento do estudo e cinco crianças (G2) não estavam e nunca haviam realizado intervenção direta/terapia fonoaudiológica. A média de idade das crianças que haviam realizado intervenção direta no início dos encontros - G1 - foi de 4,56 anos, com desvio padrão 2,10 e mediana de 3,75 anos e no final dos encontros, a média de idade foi de 4,95 anos, desvio padrão 2,06 e mediana 4,11. A média de idade do G2 no início dos encontros foi de 3,44 anos, com desvio padrão 1,58 e mediana de 3,00 anos e no final média de 3,72 anos, desvio padrão 1,70 e mediana 3,30.

Na análise dos dados demográficos e de caracterização da amostra dos participantes do G1 constatou-se que a maioria das crianças pertencia ao sexo masculino (65,4%), frequentava a educação infantil (73,1%) e apresentava diagnóstico de TEA (84,6%). Quanto aos pais, a maioria possuía ensino superior completo (76,9% e 50,0%, mães e pais, respectivamente) e metade das mães e pais estavam empregados (50,0% cada). A maioria dos cuidadores que participaram dos encontros de orientação pertencia ao sexo feminino (92,3%) e eram mães das crianças (90,3%); a maioria também possuía ensino superior completo (76,9%) e participou de todos os encontros propostos (67,7%). Os dados demográficos e de caracterização da amostra dos participantes do G2 revelaram que a maioria das crianças pertencia ao sexo masculino (80,0%), frequentava a educação infantil (60,0%) e apresentava suspeita diagnóstica de TEA (60,0%). Quanto aos pais, a maioria possuía ensino superior completo (60,0% para ambos) e estavam empregados (60,0% cada). A totalidade dos cuidadores pertencia ao sexo feminino e era mãe da criança (100,0%) e a maioria possuía ensino superior completo (60,0%) (Tabela 1).

**Tabela 1:** Análise descritiva dos dados demográficos e caracterização da amostra

**Variáveis**	**G1 - 26**				**G2 - 5**			
	**N**		**%**		**N**		**%**	
**Sexo criança**								
Feminino	9		34,6		1		20,0	
Masculino	17		65,4		4		80,0	
Total	26		100,0		5		100,0	
**Escolaridade criança**								
Não frequenta escola	3		11,5		1		20,0	
Educação infantil	19		73,1		3		60,0	
Fundamental	4		15,4		1		20,0	
Total	26		100,0		5		100,0	
**Diagnóstico TEA**								
Não	4		15,4		3		60,0	
Sim	22		84,6		2		40,0	
Total	26		100,0		5		100,0	
**Escolaridade materna**								
Fundamental Incompleto	1		3,8		-		-	
Médio completo	5		19,2		2		40,0	
Superior completo	20		76,9		3		60,0	
Total	26		100,0		5		100,0	
**Profissão materna**								
Trabalhador doméstico	8		30,8		-		-	
Autônomo	5		19,2		2		40,0	
Empregado	13		50,0		3		60,0	
Total	26		100,0		5		100,0	
**Escolaridade paterna**								
Fundamental Incompleto	1		3,8		1		20,0	
Médio incompleto	1		3,8		1		20,0	
Médio completo	8		30,8		-		-	
Superior incompleto	3		11,5		-		-	
Superior completo	13		50,0		3		60,0	
Total	26		100,0		5		100,0	
**Profissão paterna**								
Autônomo	10		38,5		2		40,0	
Empregado	13		50,0		3		60,0	
Desempregado	3		11,5					
Total	26		100,0		5		100,0	
**Sexo do cuidador**								
Masculino	2		7,7		0		0,0	
Feminino	24		92,3		5		100,0	
Total	26		100,0		5		100,0	
**Escolaridade cuidador**								
Fundamental Incompleto	1		3,8		-		-	
Médio completo	5		19,2		2		40,0	
Superior completo	20		76,9		3		60,0	
Total	26		100,0		5		100,0	
**Posição familiar cuidador**								
Mãe		28		90,3		5		100,0
Pai		2		6,5		0		0,0
Avó		1		3,2		0		0,0
Total		31		100,0		5		100,0
**Cuidador participou de todos os encontros**								
Não		10		32,3		3		60,0
Sim		21		67,7		2		40,0
Total		31		100,0		5		100,0

**Legenda:** N = número de indivíduos

Foi realizada a análise das medidas descritivas da avaliação do perfil comunicativo das crianças que estavam e não estavam em terapia direta, por meio da prova de Pragmática do ABFW, antes e após os encontros. Para as crianças do G1 – haviam realizado ou estavam em terapia fonoaudiológica, constatou-se que a média do número de atos comunicativos por minuto foi de 5,09, antes dos encontros e de 7,15 após os encontros. Quanto ao número de funções comunicativas utilizadas, a média encontrada anteriormente a realização dos encontros foi de 6,92 e após os encontros de orientação a média foi de 8,73. Contabilizou-se ainda o número de respostas - sem função comunicativa identificada - dadas pelas crianças que revelou média de 23,12 (pré-encontros de orientação) e média de 25,65 (pós-encontros de orientação). As médias do espaço comunicativo ocupado pelas crianças foram de 24,77% e 31,58%, antes e após os encontros, respectivamente. Na análise descritiva do G2 constatou-se que a média do número de atos comunicativos por minuto foi de 5,04, antes dos encontros e de 6,86 após os encontros. Quanto ao número de funções comunicativas utilizadas, não foram constatadas mudanças (média de 8,60 encontrada anteriormente e posteriormente a realização). O número de respostas - sem função comunicativa identificada - dadas pelas crianças revelou média de 28,60 (pré-encontros de orientação) e média de 18,80 (pós-encontros de orientação). As médias do espaço comunicativo ocupado por essas crianças foram de 25,60% e 40,60%, antes e após os encontros, respectivamente.

Visando completar a análise descritiva da avaliação do perfil comunicativo das crianças antes e após a intervenção (prova de Pragmática do ABFW), os seguintes dados foram reconhecidos: a maioria das crianças não possuía o número de atos comunicativos conforme o esperado para a idade^(11)^ antes dos encontros (76,9% das crianças que haviam realizado ou estavam em terapia e 80,0% das crianças que nunca haviam realizado intervenção direta). Após os encontros de orientação, a maioria dos participantes alcançou o número de atos comunicativos adequado para a idade (53,8% das crianças do G1 e 80,0% das crianças do G2). No que diz respeito aos meios comunicativos notou-se que a maioria das crianças que estavam em intervenção direta utilizava preferencialmente, no momento anterior aos encontros, o meio gestual para comunicar-se (38,5%). Em contrapartida, após os encontros, a maioria dessas crianças utilizou preferencialmente o meio verbal (48,3%) para a comunicação (Tabela 2).

**Tabela 2:** Análise descritiva da avaliação do perfil comunicativo pré e pós encontros

**Variáveis**	**G1 - 26**			**G2 - 5**					
	**Pré Pós Pré Pós**								
	**N**	**%**	**N**		**%**	**N**	**%**	**N**	**%**
**Número de atos comunicativos adequado à idade**									
Não	20	76,9	12		46,2	4	80,0	1	20,0
Sim	6	23,1	14		53,8	1	20,0	4	80,0
**Meio comunicativo mais utilizado**									
Verbal	8	30,8	11		42,3	3	60,0	4	80,0
Vocal	8	30,8	8		30,8	0	0	0	0
Gestual	10	38,4	7		26,9	2	40,0	1	20,0

**Legenda:** N = número de indivíduos

Para a análise de associação entre atos comunicativos por minuto, número de funções comunicativas utilizadas, número de respostas e percentual do espaço comunicativo utilizado, referente às crianças do G1, observa-se resultado com significância estatística antes e após encontros de orientação entre atos comunicativos por minuto (p=0,002) sendo que no momento pós o número de atos comunicativos por minuto foi maior; número de funções comunicativas utilizadas (p=0,016), com maior número de funções no momento pós-encontros; espaço comunicativo (p=0,001) sendo que no momento pós-encontros o espaço comunicativo utilizado pelas crianças foi maior. Em relação ao G2, a análise associação entre atos comunicativos por minuto, número de funções comunicativas utilizadas, número de respostas e percentual do espaço comunicativo utilizado, revelou resultado com significância estatística antes e após encontros de orientação entre atos comunicativos por minuto (p=0,042) sendo que no momento pós o número de atos comunicativos por minuto foi maior. As demais associações não apresentaram resultado com significância estatística (Tabela 3).

**Tabela 3:** Análise de associação funções comunicativas pré e pós encontros

**G1 – 26 G2 - 5**						
**Variáveis**	**Funções comunicativas**					
	**Pré**	**Pós**	**p-valor**	**Pré**	**Pós**	**p-valor**
**Atos comunicativos/minuto**						
Média±desvio padrão	5,09±2,93	7,15±2,58	0,002*	5,04±1,61	6,86±1,60	0,042*
Mediana	4,35	6,80		5,10	7,60	
**Número de funções utilizadas**						
Média±desvio padrão	6,92±2,61	8,73±2,07	0,016*	8,60±2,97	8,60±2,88	0,785
Mediana	7,00	9,00		9,00	10,00	
**Número de respostas**						
Média±desvio padrão	23,12±22,17	25,65±16,57	0,271	28,60±19,14	18,80±19,64	0,500
Mediana	15,00	21,50		31,00	11,00	
**Espaço comunicativo**						
Média±desvio padrão	24,77±9,59	31,58±7,45	0,001*	25,60±7,89	40,60±9,81	0,104
Mediana	23,50	32,00		26,00	37,00	

Teste Wilcoxon

* valor de p≤0,05

## DISCUSSÃO

Ao comparar o perfil comunicativo de crianças com Transtorno do Espectro do Autismo antes e após a realização de orientações fonoaudiológicas foi possível observar um aumento estatisticamente significativo no número de atos comunicativos por minuto, tanto para crianças em intervenção direta, quanto para crianças que nunca havia realizado terapia fonoaudiológica. Para as crianças que estavam em terapia fonoaudiológica no momento do estudo observou-se também um aumento estatisticamente significativo no número de funções comunicativas utilizadas e no espaço comunicativo ocupado, ao receber a intervenção indireta oferecida neste estudo.

O aumento no número de atos comunicativos por minuto condiz com uma melhora no perfil pragmático dos participantes e consequentemente um aperfeiçoamento na comunicação social. Esse achado corrobora outro estudo com enfoque na orientação parental que visava capacitar pais de crianças com TEA para o aproveitamento de situações cotidianas para aquisição da linguagem^(13)^. Cita-se que o aumento no número de atos comunicativos por minuto após as orientações, viabilizou o alcance do número adequado de atos comunicativos para a idade para a maioria das crianças, revelando a melhora dos prejuízos pragmáticos encontrados anteriormente à intervenção.

O aumento no número de funções comunicativas utilizadas pelas crianças que estavam em terapia fonoaudiológica após as orientações fornecidas aos cuidadores também implica em uma melhora nas habilidades pragmáticas dos participantes. Esse achado é respaldado por outro estudo^(14)^, que informa que no Transtorno do Espectro do Autismo, o prejuízo pragmático envolve desvantagens nas funções comunicativas adquiridas e utilizadas pelos indivíduos, principalmente nas funções interpessoais o que ocasiona dificuldades interativas à população. Há outro trabalho^(3)^ que corrobora o presente estudo, no qual se observou que, após um programa de orientação fonoaudiológica, as crianças com TEA aumentaram a flexibilidade no uso das funções comunicativas. Portanto, os trabalhos em questão ressaltam a importância do tratamento indireto para a variabilidade no uso das funções comunicativas da linguagem^(3)^ e consequente melhora no perfil pragmático das crianças.

Outro dado encontrado na amostra das crianças do presente estudo foi o aumento do espaço comunicativo ocupado pela criança após os encontros de orientação, aumento este estatisticamente significativo para as crianças que estavam em intervenção direta no momento do estudo. Tal dado relaciona-se intimamente com o aumento do número de atos comunicativos por minuto, assim como com a redução da monopolização da conversação, do número de comandos disparados contra a criança e do bombardeio de informações no momento pós-tratamento. Ressalta-se que as crianças que nunca estiveram em intervenção direta também aumentaram seu espaço comunicativo, apesar de tal dado não possuir significância estatística. Crianças com o desenvolvimento típico de linguagem mantém o diálogo respondendo e iniciando a conversação de maneira eficiente, sendo que raramente deixam de ocupar o espaço comunicativo, segundo estudo^(15)^, o que favorece a manutenção da interação. Infere-se que por meio dos encontros de orientação, os pais puderam compreender a importância de atentar-se à reciprocidade social e buscar a simetria do espaço comunicativo durante as interações com seus filhos, aguardando as manifestações espontâneas da criança. Em estudo anterior^(16)^ observou-se que, após orientação fonoaudiológica expondo a necessidade de aguardar as manifestações da criança e valorizar os momentos de pausa e silêncio, foi constatada melhora no desempenho linguístico entre mãe e filho. Dessa maneira, o destaque dado aos momentos de silêncio possibilitou que a interação ocorresse de maneira mais fluida, uma vez que se reduziram as perguntas objetivas e valorizou-se o interesse da criança.

A análise descritiva evidenciou questões relativas ao gênero, uma vez que a maioria das crianças do estudo pertencia ao sexo masculino. Segundo o Manual Diagnóstico e Estatístico dos Transtornos Mentais (DSM-5), o TEA é quatro vezes diagnosticado mais frequentemente em crianças do sexo masculino, corroborando a prevalência de meninos neste estudo^(1)^.

Foi possível constatar, por meio do presente estudo, que a maioria dos participantes possuía acesso à terapia fonoaudiológica direta no momento da pesquisa. Nos últimos anos, importantes conquistas em prol das pessoas com Transtorno do Espectro do Autismo, tais como a criação dos Centros de Atenção Psicossocial (equipamentos destinados ao tratamento de pessoas com autismo) foram alcançadas^(17)^. Diante disso, infere-se que o número de crianças em tratamento, incluindo o tratamento fonoaudiológico, também aumentou. Por consequente, é possível concluir que houve uma melhoria no acesso à terapia para crianças com TEA, concordando com o dado encontrado no presente trabalho.

A maioria dos cuidadores que participaram dos encontros de orientação pertencia ao sexo feminino, o que pode ser justificado pelo fato de que há uma prevalência de busca por atendimentos de saúde por mulheres ao comparar-se com o sexo masculino^(18)^. Há pesquisas que indicam que as mulheres possuem pior percepção sobre o estado de saúde e relatam mais morbidades quando comparadas aos homens, o que justifica a maior busca por cuidados em saúde^(19-21)^. Outrossim, o presente estudo mostrou que os participantes dos encontros eram, em sua maioria, mães das crianças, dado esse também encontrado em outros trabalhos envolvendo a participação de pais e cuidadores de crianças com TEA^(3,12)^. Com isso, o presente trabalho em consonância aos estudos citados, evidencia a maior participação materna nos processos terapêuticos da criança quando comparada a outros cuidadores.

É possível inferir que o fato de a maioria dos cuidadores possuir ensino superior completo e participar de todos os encontros propostos, influenciou de forma positiva no aumento do número de atos comunicativos por minuto, espaço comunicativo ocupado e número de funções comunicativas utilizadas após a intervenção. Segundo a literatura pesquisada, há estudos^(22,23)^ que sugerem que a escolaridade dos pais interfere na identificação precoce dos sinais de TEA. Ou seja, infere-se que quanto maior a escolaridade dos pais mais precocemente os sinais de TEA são observados; já o baixo nível de escolaridade paterna corrobora para um atraso no diagnóstico. Diante disso, é possível inferir ainda que com a identificação precoce dos sintomas, os cuidadores buscam tratamento também de maneira mais antecipada. Ademais, a grande maioria das crianças do estudo apresentava comprovação diagnóstica para o Transtorno do Espectro do Autismo. Esse achado relaciona-se aos estudos já citados^(22,23)^ que comprovaram que há uma identificação mais precoce dos sintomas de TEA pelos pais com maior escolaridade.

No que tange a análise descritiva dos dados clínicos, observa-se que o número de respostas dadas pelas crianças que nunca estiveram em terapia fonoaudiológica diminuiu e o aumento das respostas dadas pelas crianças que estavam em intervenção direta não foi significativo. Foram consideradas “respostas” os atos comunicativos produzidos pelas crianças que possuíam função unicamente de responder aos questionamentos e comandos fornecidos pelo interlocutor, visto que esses atos não possuíam nenhuma outra função comunicativa descrita no instrumento utilizado para análise^(11)^. A redução do número de respostas para o grupo de crianças sem intervenção direta e a associação não estatisticamente significativa encontrada para crianças em terapia fonoaudiológica direta ao comparar o número de respostas antes e após a intervenção pode ser justificada pelo fato de que os pais foram instruídos a reduzir o número de perguntas e comandos apresentados para a criança. A redução no número de perguntas e comandos objetivou possibilitar a ampliação das iniciativas comunicativas dos filhos e melhorar a simetria do espaço comunicativo. Assim, os pais foram orientados a substituírem as perguntas e comandos por comentários e nomeações. Em contrapartida, apesar da orientação citada, os pais foram instruídos também a aguardar o processamento linguístico da criança ao perguntar, para que ela elabore suas próprias respostas, evitando responder pelo filho^(16)^. Portanto, infere-se que os pais buscaram seguir ambas as instruções, o que pode ter interferido nos resultados encontrados para o “número de respostas dadas pela criança”.

No que diz respeito aos meios comunicativos da amostra total, notou-se que a maioria das crianças em terapia fonoaudiológica direta modificou seu meio utilizado preferencialmente (meio gestual predominante antes da intervenção para predomínio no uso do meio verbal pós-intervenção) apesar dessa mudança não ser estatisticamente significativa. Entretanto, o aumento no uso do meio verbal para a comunicação é de extrema valia, uma vez que indica que, após as orientações fornecidas, as crianças passaram a utilizar a fala de maneira mais acentuada para se comunicarem. O aumento do uso do meio verbal para a comunicação após orientação parental também foi observado em outro estudo, no qual os pais participantes relataram perceber que seus filhos aumentaram a verbalização tanto para expressar funções interpessoais quanto não interpessoais^(3)^.

O presente trabalho e os outros estudos citados evidenciam, portanto, a importância de incluir pais e cuidadores no processo terapêutico objetivando a melhora no desempenho linguístico da criança em contexto natural e espontâneo e consequente redução das dificuldades comunicativas dessas crianças^(24)^. Cita-se outro estudo^(25)^ que corrobora com essa afirmação, sendo que nesse notou-se que a intervenção de comunicação social mediada por pais é eficaz para reduzir os sintomas do TEA e promover efeitos duradouros na interação social entre pais e filhos. Para além, em outro trabalho^(26)^ observou-se que após uma intervenção de treinamento de pais, notaram-se melhorias nas habilidades de comunicação social tanto para as crianças com TEA quanto na competência comunicativa dos próprios pais. Notou-se, em um terceiro estudo^(27)^, que após a realização de orientações parentais houve uma melhora nas habilidades de comunicação social de crianças com Transtorno do Espectro do Autismo, corroborando mais uma vez os benefícios da intervenção indireta no tratamento dessas crianças.

O atual estudo, tornou evidente que a intervenção indireta associada a terapia fonoaudiológica direta traz maiores benefícios à comunicação das crianças com Transtorno do Espectro do Autismo, quando comparado aos benefícios da intervenção indireta isolada. Tal achado corrobora com outro estudo^(28)^ que evidenciou que o fonoaudiólogo, ao intervir diretamente e indiretamente com indivíduos com TEA, ocasiona evoluções mais amplas no desenvolvimento linguístico dessas crianças, uma vez que a informação direcionada aos pais proporciona um melhor acompanhamento de crianças com TEA.

A principal limitação do estudo refere-se ao número de participantes ao início e ao final dos encontros de orientação. No início do estudo, 149 pais manifestaram interesse em receber as orientações assinando o Termo de Consentimento Livre e Esclarecido fornecido de maneira remota. 98 cuidadores preencheram o questionário de caracterização da amostra e história clínica e enviaram o registro em áudio e vídeo. Desses, sete não participaram de nenhum dos encontros de orientações. Ressalta-se que dos cuidadores que participaram de quatro ou mais encontros de orientação, oito não enviaram o registro em áudio e vídeo para análise final, o que consequentemente reduziu o número de dados utilizados na amostra. Diante disso, conclui-se que 68,3% dos participantes não participaram da amostra final do estudo, 7,1% não participou de nenhum dos encontros de orientação e 8,1% participaram dos encontros, mas não enviaram o registro final. Conclui-se, portanto, que 53% dos cuidadores optaram por deixar o estudo sem justificativa prévia. O tempo determinado para realização dos encontros mensais (duas horas por encontro) pode ter influenciado de maneira negativa a participação dos pais até o final do estudo.

Além disso, o fato da amostra ter sido constituída por conveniência e a heterogeneidade dos grupos impede a generalização dos achados para outros contextos e impossibilita a comparação entre crianças que estavam e não estavam em terapia fonoaudiológica. Sugere-se, portanto, a ampliação do estudo visando alcançar mais cuidadores e providenciar um grupo comparativo e solucionar as limitações citadas.

Como avanço obtido com o estudo, cita-se a participação ativa da família no processo de desenvolvimento das crianças com TEA. O treinamento de pais por meio de orientações dos profissionais é um importante meio de aperfeiçoamento das habilidades linguísticas das crianças que com Transtorno do Espectro do Autismo, uma vez que são os pais que passam maior tempo com essas crianças e, portanto, possuem maiores chances de estimulá-las em contexto natural e espontâneo.

Além disso, o presente estudo trouxe aos pais uma visão pragmática que possui enfoque não apenas no uso de perguntas e respostas durante a comunicação, mas também no uso de outras funções comunicativas para iniciar e manter interações de maneira funcional. Acrescenta-se ainda que o subsistema pragmático da linguagem deve ser o foco da terapia fonoaudiológica voltada para os indivíduos com Transtorno do Espectro do Autismo, posto que é no uso da linguagem que os sujeitos conseguem manter a conversação e realizar o intercâmbio de informações.

## CONCLUSÃO

Conclui-se que, ao comparar o perfil comunicativo da amostra de crianças antes e após as orientações, houve mudanças considerando o aumento estatisticamente significativo no número de atos comunicativos por minuto. Ademais, foram constatados aumento estatisticamente significativo no número de funções comunicativas utilizadas e no espaço comunicativo ocupado pelas crianças que estavam em terapia direta, após a intervenção indireta descrita nesse estudo, evidenciando os aprimoramentos na comunicação social dos participantes. Desta forma, afirma-se que a realização de orientações fonoaudiológicas (tratamento indireto) mostra-se como uma boa ferramenta de intervenção que possibilita benefícios para a melhora das habilidades pragmáticas de crianças com Transtorno do Espectro do Autismo.
